# Agentic AI for Child Welfare Intake and Early Risk Identification: A Governance-Aware Framework

**DOI:** 10.7759/cureus.112295

**Published:** 2026-07-08

**Authors:** Ambar Nath Saha, Debashis Patra, Arumugam Muthu

**Affiliations:** 1 Artificial Intelligence in Healthcare, Independent Research, Halifax, CAN; 2 Information Technology, Cognizant Technology Solutions Canada, Inc., Halifax, CAN; 3 Artificial Intelligence and Machine Learning, The University of Texas at Austin, Austin, USA; 4 Information Technology, Triwave Solutions Inc., Columbia, USA

**Keywords:** agentic ai, ai governance, child welfare, conversational intake, decision support system, human-in-the-loop technology, multi-agent systems, risk assessment

## Abstract

Child welfare intake is the first point where someone raises a concern about a child, and the system receives the data and decides what to do next. Child welfare intake is where everything begins. The first few decisions taken here often shape the entire journey of a case. But in reality, this stage is messy, information comes in bits and pieces, decisions depend on individual judgment, and important details are sometimes missed. When this happens, vulnerable children may not receive the right attention at the right time.

In this paper, we present a governance-aware conceptual framework and proof-of-concept implementation of a multi-agent artificial intelligence (AI) system designed to support child welfare intake and early risk identification. We have envisioned a system where the data entry will be done using a conversational interactive chatbot interface, and the system will have multiple agents to perform specific tasks such as analyzing the data, assessing risk, checking data quality, and monitoring bias. Also, there will be a Central Orchestrator, which will manage all the agents and maintain the sequence of the workflow.

A couple of important ideas we've planted in our proposed system are that if the system is not confident about the output it generates, it does not propagate to the next phase; instead, it passes the case to a human in the loop. Also, the system asks human-friendly questions if the response from the user is incomplete. These features make the system more thoughtful rather than just rushed, and they help ensure that uncertain or high-risk situations are not handled only by automation.

Our intention through this article is not to reduce human involvement in the child welfare system or to take over the role of caseworkers. Our main goal is to propose an agentic AI-enabled support system for caseworkers that has the potential to improve the consistency, completeness, and governance of child welfare intake processes. With appropriate real-world validation, the proposed system may play a supportive role in helping vulnerable children receive attention earlier.

## Introduction

Child welfare systems handle a huge number of intake cases every year [1]. Unfortunately, most systems receive either non-structured data or incomplete information. Also, intake workers are often forced to make rushed decisions due to a huge workload [2]. This is the stage, and the most crucial stage, of the entire child welfare lifecycle where case details are first captured, and the system judges the initial risk, and eventually decisions are made on what to do next. It is a paramount responsibility for the system or caseworkers to make things right at this stage, as if something goes wrong at the beginning, it will have a snowball effect down the line.

We should not think of the intake process as just an administrative step from a child welfare perspective. It is the first step toward providing a better and safer future for all vulnerable children. If the intake process is incomplete or the risk is not analyzed at the initial level, then it will have a chain effect on the entire process, and as a result, those children or families may experience delays in receiving proper support. This is really important, especially for children with disabilities or children living in unsafe and unstable environments. So, we are not solving just a technical problem; instead, we are thinking about child safety and trying to address a social responsibility problem.

Today, many systems still depend on manual legacy data entry styles with rule-based tools where all reporters have to fill out a big form, and then the data will be entered into the system, and they struggle when the data is incomplete and unclear [1]. Nowadays, many artificial intelligence (AI)-enabled systems are built, but in most cases, they are using only a one-model concept, and that's where the system starts to break [3-5].

Prior child welfare decision-support approaches have usually focused on structured decision-making, rule-based workflows, or centralized predictive scoring. In high-risk intake settings, a single-model approach may not be enough because intake requires several separate tasks, including understanding unstructured information, identifying missing details, assessing risk, checking for bias, explaining the outcome, and escalating uncertainty. Our proposed framework takes a different direction by separating these responsibilities across specialized agents and connecting them through a governance-aware workflow with confidence-based abstention, audit logging, and human review.

We have conceptualized an agentic AI system consisting of multiple agents, where each agent is specialized to perform a specific task and will have a clear role in the entire workflow [6]. There will be a Central Orchestrator, who is going to govern the entire workflow and manage all those agents [7]. Our goal is to build a governance-aware agentic AI intake system where most of the repetitive work will be done by agents while keeping all the governance validations intact, and also a human checkpoint should be present when the system itself is unable to take any decision [8]. Here are the different agents along with their responsibilities: Intake Understanding Agent (IUA) to interpret the input [9]; Risk Scoring Agent (RSA) to assess the level of risk [10]; Data Quality Agent (DQA) to check for missing or inconsistent information; Bias Monitoring Agent (BMA) to keep fairness in check [11]; and Explanation Agent (EA) to present the outcome in a human-readable way [12].

The primary objective of this article is to present a governance-aware multi-agent framework for child welfare intake and assess its technical feasibility through a proof-of-concept prototype in a controlled environment, with emphasis on conversational intake, specialized agent responsibilities, confidence-based abstention, auditability, and human review.

## Materials and methods

For clarity, the methodology is presented in three parts: first, the design of the proposed governance-aware multi-agent intake workflow; second, the proof-of-concept prototype implementation; and third, the controlled evaluation approach using synthetic intake scenarios to verify workflow behavior, escalation logic, and auditability.

Challenges in current intake systems

The following are a couple of highlighted challenges that we have experienced in our current intake system:

Inconsistent Data Capture

Most of the systems have a manual data entry process, which is a bit messy and time-consuming affair. Some organizations have built online data entry systems, but they usually follow form-based data entry, which struggles in collecting any unstructured data, such as information from phone calls or written narratives [2]. 

Incomplete Information

Many intake reports do not have all the required details, which makes it difficult to make the right decision at the right time [4]. In child welfare, incomplete information is not only a documentation issue. It may delay the identification of risk and may prevent a child or family from receiving timely support.

Subjective Risk Assessment

We have to agree on one point that most of the caseworkers are experienced and have adequate skills to assess any risk [13], but as the risk assessment is totally dependent on one person's expertise and observation, it's highly likely they may see the same situation differently based on their prior experience, circumstances, workload, or personal bias [3]. This variation can become more serious when the child is very young, has a disability, or belongs to a family already facing social or economic stress. 

Early-Stage Bias

Any form-based system doesn't have the capability to sense any bias, and it's difficult and time-consuming for any caseworker to identify bias in the workflow. It's very crucial to identify and mitigate bias at this very early stage, as it impacts all the later decisions [1,8].

Lack of Scalability

The scalability of the system is dependent on how many caseworker resources are available. This legacy and manual system doesn't have the capability to scale up if the number of cases increases [6].

Proposed solution: a governance-aware multi-agent intake workflow

We propose a governance-aware multi-agent workflow to collect all the key information about a child in a more efficient way, keep a consistent risk management agent along with a human-in-the-loop, automate a couple of monotonous tasks, make the process more consistent, and help caseworkers focus on truly complex work while keeping governance checks intact to make sure the child's data is secured [7].

The workflow is built on three main ideas: conversational intake, clear agent responsibility, and human oversight. Our system proposes a conversational intake interface instead of a rigid form-based interface, where the reporter can write about the child in an unstructured way and can also upload documents, portable document format (PDFs), audio, video, etc. Our system has multiple agents that take care of specific responsibilities separately, and all those agents are going to take that unstructured data and, through an entire workflow, create a structured output for the caseworker [9]. Multiple agents are needed to keep the governance checks intact and make the system scalable and auditable. Human oversight is very important in this type of autonomous system, and every agent works based on confidence level [4]. If the confidence level is lower than the threshold value, then the system abstains from taking any decision, and the case will be redirected to a human for review [10]. All the threshold values are basically configurable values, which will be determined during actual product implementation.

End-to-end intake workflow

The workflow begins when a reporter or intake worker opens our chatbot-type interface to enter any child's information. The system immediately creates an intake record with the available data that is shared by the user. The system has weightage-based fuzzy search logic to check for any duplicate records, and if the system is very confident (the threshold value of the confidence is a configurable percentage) about the existing record matching, then instead of creating a new intake, it updates the existing record. Sometimes, the system asks the reporter whether the person is referring to the same child who is already present in the system's record.

The user can enter any type of unstructured textual data, or they can upload any document, videos, or photographs of the child. The IUA will analyze all the unstructured data, extract all the important information, and try to prepare a structured dataset [6,14].

As our proposed intake interface is conversational, the system does not silently enter the data if any critical data is missing. Instead, it creates a human-friendly question and asks the reporter to provide the data. This is important because reporters may not always know which details are important during the intake process. A conversational follow-up flow can help collect missing information without making the reporter feel overwhelmed by a long form. After a couple of follow-up questions, if the system still cannot collect all the relevant and critical information, the case is immediately redirected to a human for review. The system has a limited follow-up loop, and it is designed not to overwhelm the user. This loop limit is configurable.

Once the system collects most of the important data after the conversational loop, the RSA starts assessing the child's vulnerability. It analyzes many different scenarios, such as the child's age, security concerns, any noticeable injury, special needs, prior history, etc., based on the available data and generates a risk score. If the risk is very high and above a system-configured threshold value, the caseworker gets immediately notified about the situation so that they can make further decisions. The purpose of this early notification is to reduce delay in situations where the child may need urgent attention.

After the Risk Assessment, the DQA mainly checks the data quality before it goes for caseworker review. We have kept this agent in the loop to avoid any unnecessary delay because of incomplete data. It checks whether all the required and crucial data is present or not, and if the data is not present, then it again sends a signal to the first agent to ask for that information.

The main responsibility of the BMA is to check whether the risk assessment is fair and unbiased. There could be multiple unfair factors, such as demographic bias, location-based bias, and socioeconomic biases, that can influence the system's output, and similar cases can be treated differently. If this agent finds any discrepancies, then it redirects the case to humans for review.

Once all the checks are completed, the system saves the intake record along with the raw narrative, uploaded file references, extracted fields, follow-up history, risk score, data quality result, bias monitoring result, confidence scores, and audit events. After that, the EA converts all the technical outputs into a simple summary for the caseworker. The summary includes the risk level, confidence, urgency, key reasons, limitations, and recommended next step.

**Figure 1:**
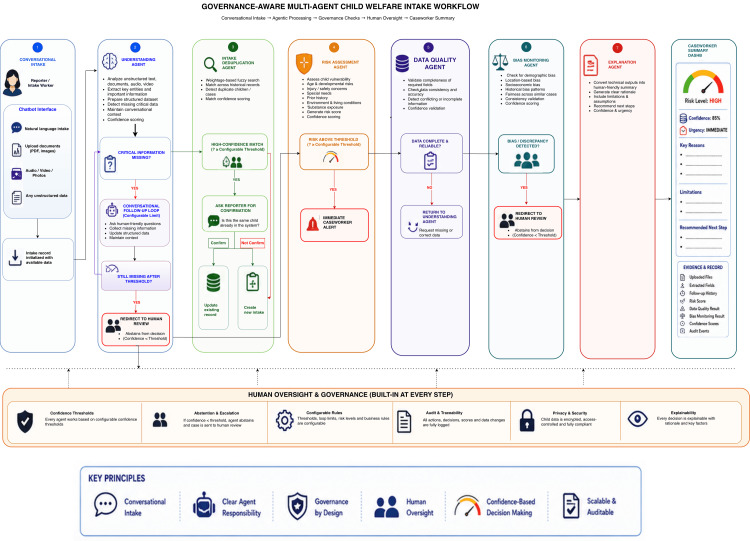
Governance-aware multi-agent child welfare intake workflow. The proposed framework separates responsibilities across conversational intake, orchestration, risk assessment, governance validation, human oversight, and persistence layers. The architecture emphasizes modularity, auditability, configurable decision thresholds, and human-in-the-loop escalation for uncertain or high-risk scenarios. The figure illustrates how unstructured intake information is transformed into structured decision-support artifacts while maintaining governance and traceability. The authors created the figure using diagrams.net (formerly draw.io), developed by JGraph Ltd., London, United Kingdom. The editable source files for the manuscript figures are provided in Appendix 6.

The complete governance-aware intake workflow is shown in Figure 1. It summarizes how the system moves from conversational intake to intake understanding, duplicate checking, risk assessment, data quality review, bias monitoring, explanation generation, and caseworker-facing summary. The same figure also shows how human oversight, confidence thresholds, auditability, privacy, and explainability are built into the workflow. A more detailed version of the business workflow is provided in Appendix 2.

Agent responsibilities

We have proposed a multi-agent framework where each agent is designed to perform a specific role. There will be a governance-aware Orchestrator that coordinates the execution sequence of each agent. Another important responsibility of the Orchestrator is to enforce the confidence threshold of each agent's output, and it determines when to escalate to a human reviewer if the output doesn't meet the confidence threshold. The Orchestrator is implemented using LangGraph (LangChain, Inc., San Francisco, CA, USA)/StateGraph, where all the agent logic is executed as part of a controlled workflow. The agents use Model Context Protocol (MCP) servers to interact with backend services and databases, which helps keep the system modular, traceable, and governance-aware. 

Agent 1: Intake Understanding Agent (IUA)

This agent reads the information submitted by the reporter from the React/Vite frontend. The input can be a chatbot conversational message or uploaded files such as documents, images, videos, or other attachments. The FastAPI backend receives the request and sends it into the LangGraph/StateGraph workflow.

This agent extracts information from all those unstructured records with the help of the large language model (LLM) layer through the shared LLM factory, which is connected with Claude Opus through the Anthropic Application Programming Interface (API) (Anthropic PBC, San Francisco, CA, USA). It uses multiple specific MCP servers to complete a couple of tasks, such as fuzzy search, file uploading, and inserting intake information, etc.

Agent 2: Risk Scoring Agent (RSA)

The RSA, which runs inside the LangGraph/StateGraph workflow, evaluates the structured inputs and looks for any vulnerability, such as whether the child is young, has some visible injuries, is starving, etc. Based on the severity level, it assigns a risk level: low, medium, high, or critical, along with a confidence score and contributing factors. This agent may use MCP servers to check any previous case history, if required, to increase the confidence of the output of this agent.

In the proof-of-concept, the RSA uses predefined risk-factor categories to demonstrate decision routing rather than a validated predictive model. The factors include age-related vulnerability, visible injury or safety concern, reported neglect or unsafe environment, disability or special needs, prior case history, urgency of the reported concern, and completeness of the available intake information. The agent assigns a preliminary risk category based on the presence and severity of these factors and then sends the result, confidence level, and contributing reasons to the Orchestrator. In a production implementation, this decision logic would need to be replaced or calibrated using trained domain-specific models, real-world child welfare data, and expert validation.

Agent 3: Data Quality Agent (DQA)

This agent checks whether the intake data is clear and complete enough for the caseworker review. For example, if any information is required or a critical field is missing in the input request, this agent is going to generate follow-up questions and send those questions to the conversation frontend via the FastAPI backend. This agent is important because missing or unclear information can delay caseworker review and may slow down support for the child or family.

Agent 4: Bias Monitoring Agent (BMA)

The primary responsibility of this agent is to check fairness across demographic groups. If the agent finds any anomalies, then it does not move the case further; instead, it sends the case to the caseworker for manual review. Also, it keeps all the audits and logs so that caseworkers can review the output from logs and audits.

In the proof-of-concept, the BMA checks whether similar intake scenarios receive different risk outputs when fairness-sensitive factors such as demographic background, location, disability status, or socioeconomic context are changed. If the risk category or explanation changes without a clear case-specific reason, the case is flagged for human review. These checks are used to demonstrate governance routing only and are not presented as a validated fairness framework. In a production system, fairness metrics and thresholds would need to be defined with domain experts and validated using real-world child welfare data.

Agent 5: Explanation Agent (EA)

This agent converts all the technical outputs into human-readable structured output with the help of the LLM layer through the LLM factory, which includes risk level, reasoning, confidence scores, data completeness, etc.

The final summary is shown in the caseworker/reviewer interface, which includes the risk level, main reasons, limitations, missing information, etc. These records help caseworkers quickly understand why a case was flagged, what information is missing, and what limitations exist in the system output. The complete business requirement structure used for the prototype is provided in Appendix 3.

Human-in-the-loop decision gates

Human checkpoints are crucial in making any autonomous work properly, and we have kept human checkpoints at each decision-making point. Every agent generates output along with a confidence level, and if any agent is not confident enough to make a decision, then it will be sent for human review. We have used multiple threshold values to determine when agents should reach out to humans, but as this is a proposed and conceptual system, we haven't specified values for those thresholds. Those threshold values can be determined during actual product development.

The human-in-the-loop in child welfare is essential, as we are dealing with a child’s future here. We cannot let the system decide the future of a child, especially when the system is uncertain or does not have enough confidence in the output. If the information is unclear, incomplete, or sensitive, the case should move to a human reviewer instead of continuing through automation.

**Figure 2:**
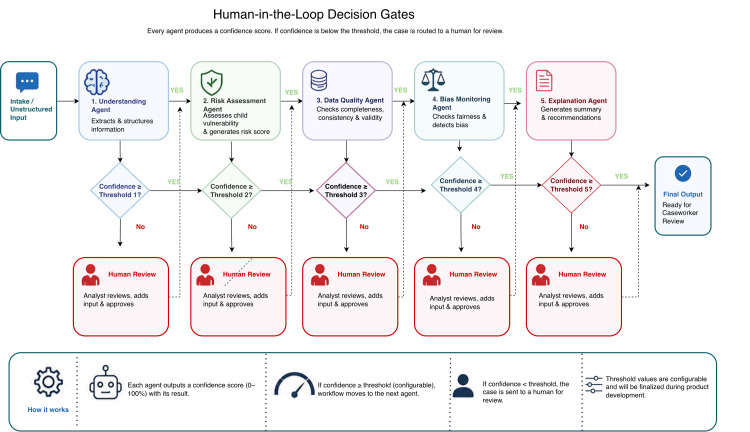
Confidence-aware abstention and human-in-the-loop escalation mechanism. The orchestration framework continuously evaluates agent confidence scores during workflow execution. If confidence falls below configurable governance thresholds, automated progression is halted, and the case is escalated for human review. This mechanism reduces the risk of unreliable autonomous decisions in sensitive child welfare scenarios and reinforces responsible AI governance practices. The authors created the figure using diagrams.net (formerly draw.io), developed by JGraph Ltd., London, United Kingdom.

The confidence-aware human review flow is illustrated in Figure 2. The figure shows how each agent produces an output with a confidence score and how the workflow is interrupted when the score falls below the configured threshold. In such cases, the intake is redirected to a human reviewer instead of continuing automatically.

Technical architecture and prototype implementation 

Although in this paper we mainly worked on conceptualizing the framework, we also built a proof-of-concept prototype. The frontend was developed using React/Vite, where reporters can submit intake information through a conversational screen and upload documents, images, videos, or other files. The prototype has another module for caseworkers where all the cases will be listed so that caseworkers can review those intakes and they can approve or escalate based on their observation.

The backend was built using FastAPI. It has separate services for all different tasks, such as intake submission, case review, follow-up questions, audit logging, notifications, reporting, etc.

The central orchestration layer was implemented using LangGraph/StateGraph. This layer controls the full workflow and decides which agent should run next. All agents use a shared LLM layer through the LLM factory, which is connected with Claude Opus through the Anthropic API. We used this shared LLM factory so that model access, prompts, and settings can be managed from one place instead of handling them separately inside each agent.

One of the important architectural decisions that came out during the development phase is the use of MCP servers. Instead of each agent connecting directly to databases or backend services, all are routed through dedicated MCP servers. We have used the MCP servers to make our system modular and governance-aware.

We used Amazon DynamoDB and Amazon Simple Storage Service (Amazon S3) (Amazon Web Services, Inc., Seattle, WA, USA) for data persistence. Amazon DynamoDB stores structured data, such as case history information and audit records, whereas documents, audio files, and videos are stored in Amazon S3. Because deploying the entire prototype in a live Amazon Web Services (AWS) (Amazon Web Services, Inc., Seattle, WA, USA) environment would incur substantial costs, we used LocalStack (LocalStack GmbH, Zurich, Switzerland) within a local Docker (Docker Inc., Palo Alto, CA, USA) container to emulate AWS services on a local machine. This approach enabled cost-effective development and testing of the prototype while maintaining compatibility with the target AWS environment.

All the threshold values are going to be configured with the help of environment variables. We have used Pydantic models for schema validation to keep consistency across APIs, and observability and monitoring are handled by CloudWatch-style logging and monitoring.

This is a very important point to note down: in this proof-of-concept implementation, we have used the shared LLMs, but the structure would differ when we plan to build this system for real-world usage. In this article, we concentrated on building the entire system where governance validations, audits, modularization, and interactive approaches with reporters should be taken care of with the help of agentic AI, and that's the reason we have limited our research to conceptualizing the entire system rather than training our own models. But in the future, we'll train domain-specific models with real-world datasets to get deterministic outputs, and deterministic models should be present along with LLMs. Also, all the threshold values mentioned in this paper will be determined after training those deterministic domain models.

**Figure 3:**
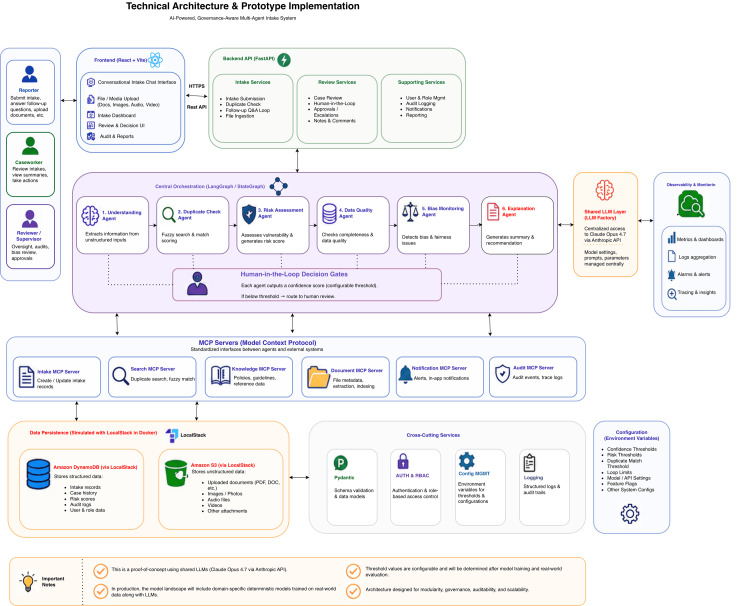
Prototype technical architecture for governance-aware child welfare intake orchestration. The proof-of-concept implementation combines a React/Vite conversational interface, FastAPI backend services, LangGraph/StateGraph orchestration, MCP-based service integration, and cloud-native persistence components. Structured and unstructured intake data are processed through modular agent workflows while maintaining auditability, configurable governance policies, and human-review escalation mechanisms. Amazon DynamoDB and Amazon Simple Storage Service (Amazon S3) (Amazon Web Services, Inc., Seattle, WA, USA); LocalStack (LocalStack GmbH, Zurich, Switzerland); Docker (Docker Inc., Palo Alto, CA, USA) The authors created the figure using diagrams.net (formerly draw.io), developed by JGraph Ltd., London, United Kingdom.

The technical architecture of the proof-of-concept prototype is shown in Figure 3. It shows how the React/Vite frontend, FastAPI backend, LangGraph/StateGraph orchestration layer, shared LLM factory, MCP servers, DynamoDB, S3, LocalStack, and monitoring components are connected together. Additional technical requirements and implementation details are provided in Appendix 4.

The “I don’t know” mechanism

In a sensitive area like child welfare, we should not give a provision to an automated system to generate any output without any guardrails because any uncertain output would be harmful if that becomes the final decision without any checks. So, this mechanism is not just a technical feature but also an ethical safeguard.

A critical feature and the most responsible feature of the system is to abstain from making any automated decision when the system's confidence is insufficient. Each agent is supposed to produce confidence scores, and the Orchestrator, which is built using LangGraph/StateGraph, monitors the confidence score in every flow [5,15]. If somehow the confidence score is below the threshold, the flow will be interrupted, and the case will be routed to a human reviewer through the FastAPI review services. This methodology guarantees that although the system is autonomous, human checks are an integral part of the system [4]. This article is not proposing any fixed threshold value; rather, the thresholds are dynamically adjusted based on risk severity and model training [10]. 

In a production implementation, the confidence score would be generated after training and validating domain-specific models using real-world child welfare data. The trained model output would then be calibrated through testing, expert review, and validation against known intake outcomes so that the confidence score reflects how reliable the model is for a specific task. For example, the RSAs confidence would depend on how strongly the trained model supports a risk category based on available risk factors, while the IUAs and DQAs would use confidence estimates based on field extraction quality, completeness, consistency, and validation results. Since the current study is a conceptual proof of concept and does not include real-world model training, the threshold values used here are illustrative and configurable design assumptions rather than empirically derived production values.

Every agent produces a confidence score between 0 and 1 [10]. If the score falls below a threshold, the system stops processing and redirects the case for human review. The thresholds are stricter for higher-risk cases, and the illustrative threshold values used in the prototype are shown in Table 1:

**Table 1:** Illustrative confidence thresholds used by the governance-aware multi-agent workflow for autonomous child welfare intake processing. Thresholds are dynamically adjusted according to case risk severity. If an agent’s confidence score falls below the configured threshold, the orchestration workflow interrupts automated processing and routes the case for mandatory human review. The Bias Monitoring Agent maintains a consistently high threshold across all risk categories to enforce fairness and governance safeguards. These threshold values are prototype-level examples intended to demonstrate abstention and escalation behavior and are not clinically or operationally validated for production deployment.

Agent	Low Risk	Medium	High	Critical
Intake Understanding	0.60	0.70	0.80	0.90
Risk Scoring	0.65	0.75	0.85	0.90
Data Quality	0.70	0.80	0.85	0.90
Bias Monitoring	0.95	0.95	0.95	0.95

The BMA uses a consistently high illustrative threshold across all risk levels to reflect the importance of fairness checks in this proof-of-concept workflow [1,8]. This threshold is implemented in the LangGraph/StateGraph workflow as a conditional routing step that either moves the case to the next agent or sends it to the human review flow [5]. These thresholds were selected as configurable design assumptions for prototype demonstration and were not derived from empirical testing or expert consensus. In a production implementation, they would need to be calibrated and validated using real-world child welfare data and domain expert review before operational use.

## Results

As part of the proof-of-concept implementation, we tested the proposed framework using multiple controlled intake scenarios and autonomous Software Development Life Cycle (SDLC)-driven testing workflows. Since the goal of this study was to assess governance-aware workflow feasibility and system orchestration rather than train or validate a production-ready predictive model, we focused on workflow-level functional validation instead of traditional machine learning metrics such as precision, recall, or F1-score.

The prototype was tested across multiple areas such as end-to-end workflow execution, follow-up question generation, duplicate detection, escalation handling, audit generation, and frontend-backend integration. More than 30 synthetic test scenarios were autonomously generated [16,17] and executed through testing agents using different combinations of complete information, incomplete information, duplicate records, high-risk situations, and fairness-related edge cases. The detailed test requirements, validation structure, and proof-of-concept test artifacts are provided in Appendix 1.

Testing showed the Orchestration layer, which is built using LangGraph, was able to coordinate multiple agents as expected. The system was able to convert unstructured data into structured data and was able to save the data into DynamoDB. We also tested the same flow with incomplete data, and the system came back to the reporter and asked them to provide the critical information in a human-friendly manner. The weightage-based fuzzy search for duplicate records worked very well with different confidence levels. We have checked the human-in-the-loop checkpoints and observed that agents were redirecting the case for human review if the confidence was low.

In this proof of concept, we have defined success based on whether the system has completed the expected workflow or not. For example, if the data entered by the reporter is incomplete, then we check whether the system is generating human-friendly follow-up questions or not. We created such test cases to verify whether the system is responding in the same manner as expected. 

Overall, the prototype has shown promising output, and it has supported our concept of building a governance-aware agentic AI child welfare intake framework. But at the same time, we need to keep in mind that the entire system was tested based on synthetic data and also in a controlled environment using shared LLM-based reasoning, and did not validate real-world child welfare outcomes. In the future, we plan to train domain-specific deterministic models using real-world child welfare data and validate the system with actual caseworkers and domain professionals. A demonstration video of the proof-of-concept workflow is provided in Appendix 5.

## Discussion

The proposed agentic AI framework may be safer than a single-model approach

Compared with structured decision-making tools, rule-based intake workflows, and centralized predictive risk models, the proposed framework separates intake understanding, risk scoring, data quality review, bias monitoring, explanation, and human escalation into distinct, auditable steps [2,13].

A single-model system behaves like a black box, and we don't always get to see the reason when something fails [11,12]. In our proposed framework, each agent handles specific work, and every step is properly logged for traceability [9,15]. Our system abstains from providing any output or interrupts the autonomous process when it finds that the confidence is low or any bias is detected [8,10]. The human checkpoint is in place to review any uncertainty [4].

Ethical guardrails

Our system is designed to support caseworkers, not to replace them [12]. Instead of doing all the monotonous jobs, they now focus on solving some complicated and uncertain cases where the system is not able to make any decision on its own. The proposed system will work hand in hand with case workers to make the processes faster and smoother [2,13].

Potential value for children and families

The proposed framework may support children and families by improving the quality of early intake information and helping urgent concerns reach caseworkers faster. Children or families in high-volume environments may experience delays due to manual processes. If validated in real-world settings, a framework like this may help caseworkers review intake information more consistently and identify cases that require timely attention [2,13].

Traceability

Another important point is traceability. The system not only processes the intake but also moves forward. It also keeps the history of what happened in each step. The system logs each and every step, such as user input, any conversation between the system and user, all the agents’ activities, confidence scores at each level, etc., which helps caseworkers understand why a case was escalated or why it was kept for review [7,11,15].

Limitations and future work

There are a couple of clear limitations that we acknowledge and make sure are explicitly mentioned in the paper. In this article, we have conceptualized an agentic AI-enabled system, but how it will interact with real-world settings is totally unknown to us. Although we have tested all the governance validations and human checkpoints and have received very promising results, this study did not use real-world child welfare datasets. The success of the system is totally dependent on model training with real data [1,8]. The synthetic scenarios were internally generated and may not represent all real-world intake patterns. The proof-of-concept does not include adversarial testing, stress testing, or independent scenario design. We have introduced bias checking, but the efficiency of the BMA totally relies upon how all the models are trained with diverse data [1,3,8]. Running multiple agents is definitely going to improve control and reliability, but it also comes with a higher compute cost [15]. The prototype was not evaluated by actual caseworkers or social workers. We have yet to explore whether caseworkers would accept this tool as part of their regular support system. 

This study also did not measure real-world outcomes such as reduced response time, improved service referral, or better child safety outcomes [13]. The proof-of-concept does not perform online learning during runtime. Intake records and audit logs are stored for review, but model behavior is not updated automatically from live intake submissions.

As this paper is all about conceptualizing an agent AI framework to support the intake process, we have not done any model training, and we used LLMs for the prototype [5,14,15]. Because the proof-of-concept uses shared LLMs, the system may also be affected by hallucinations, output variability, prompt sensitivity, and reproducibility challenges; therefore, any production use would require stronger validation, deterministic safeguards, and domain-specific model calibration. The proof-of-concept implementation artifacts, workflow specifications, and technical documentation are publicly available in the project repository [18].

## Conclusions

A child welfare system is an integral part of society, and its success depends on how efficient the intake process is. So, it is time that we look beyond the manual legacy process and think of building an AI-enabled system. But it is too sensitive to rely on just a black-box AI without any governance framework. This framework takes a middle path: specialized agents, clear governance, and deployable cloud-ready infrastructure.

Controlled proof-of-concept testing showed that the framework can support structured intake capture, follow-up questioning, human escalation, auditability, and bias-aware review. But along with efficiency, we propose an ethical and responsible framework that knows when to step back and let a human decide. In this space, getting intake right means help reaches faster. That’s the goal.

## Appendices

Appendix 1

System Specification and Test Cases

Appendix 1 provides the detailed system specification for the proposed Agentic AI Child Welfare Intake proof of concept. It includes the requirement traceability structure, business requirements, functional requirements, agent requirements, human-in-the-loop requirements, API requirements, database requirements, non-functional requirements, LangGraph workflow requirements, deployment requirements, and test requirements. It also documents how the system supports traceability from business goals to implementation and validation artifacts.

https://github.com/adnimbuslab/Agentic_AI_Child_Welfare_Intake/blob/main/SPEC.md   

Appendix 2

Business Workflow

The following business workflow document describes the end-to-end child welfare intake workflow. It explains how the system supports conversational intake, document upload, structured field extraction, duplicate detection, follow-up questions, risk assessment, data quality review, bias monitoring, human escalation, and caseworker-facing summaries.

https://github.com/adnimbuslab/Agentic_AI_Child_Welfare_Intake/blob/main/Business_Workflow.md 

Appendix 3

Business Requirements

Appendix 3 defines the business and functional requirements for the proposed system. It describes the intended users, including reporters, caseworkers, and supervisors, and explains how unstructured intake narratives and supporting documents are converted into structured data for review. It also covers risk scoring, missing information handling, data quality validation, bias monitoring, and human oversight.

https://github.com/adnimbuslab/Agentic_AI_Child_Welfare_Intake/blob/main/Business_requirement.md  

Appendix 4

Technical Requirements

Appendix 4 provides the technical high-level design for the proof of concept. It describes the proposed React frontend, API Gateway, Lambda-based backend, DynamoDB data model, LangGraph-based agent workflow, MCP server boundaries, audit logging, and caseworker dashboard. It also defines the responsibilities of the Intake Understanding, Risk Assessment, Data Quality, Bias Monitoring, and Explanation agents.

https://github.com/adnimbuslab/Agentic_AI_Child_Welfare_Intake/blob/main/technical_requirement.md 

Appendix 5

Demo video https://github.com/adnimbuslab/Agentic_AI_Child_Welfare_Intake/blob/main/demo_10_min_walkthrough.mp4

Appendix 6

Figure Source Files

All figures and architectural diagrams included in this manuscript were manually designed by the authors using standard diagramming and visualization tools. No AI-generated images or synthetic artwork were used in the preparation of the manuscript figures.

https://github.com/adnimbuslab/Agentic_AI_Child_Welfare_Intake/blob/main/workflow%20(1).drawio

https://github.com/adnimbuslab/Agentic_AI_Child_Welfare_Intake/blob/main/humen%20in%20loop.drawio%202

https://github.com/adnimbuslab/Agentic_AI_Child_Welfare_Intake/blob/main/architecture.drawio
